# Supplementary material for: Pin-Pointing the Key Hubs in the IFN-γ Pathway Responding to SARS-CoV-2 Infection
Source: Viruses. 2022 Sep 30;14(10):2180. doi: 10.3390/v14102180 (PMC9610092; doi:10.3390/v14102180)
Supplement: Supplementary file 1 [file viruses-14-02180-s001.zip › viruses-1862194-supplementary.pdf]

Supplementary information

Supplementary Table S1. Patient demographics for the GSE152075 dataset.

		non-COVID-19	COVID-19	*p*-value
**Number of patients**		50	403	
**Sex**	**Male**	22 (44%)	166 (41.2%)	
	**Female**	28 (56%)	187 (46.4%)	0.763 [a]
	**Unknown**	-	50 (12.4%)	
**Age (years)**	**Range**	12 - 91	2 - 91	
	**InterQuartile Range**	29-63	41-71	
	**Median**	46.5	56	
	**Media**	46.5	55.6	0.002 [b]
	**<30s**	13 (26%)	41 (10.2%)	
	**30s**	5 (10%)	51 (12.7%)	
	**40s**	10 (20%)	55 (13.6%)	
	**50s**	7 (14%)	80 (19.9%)	
	**60s**	8 (16%)	50 (12.4%)	
	**≥70**	7 (14%)	110 (27.3%)	0.012 [c]
	**Unknown**	-	16 (3.9%)	
**Viral load**	**High (Ct <19)**	-	106 (26.3%)	
	**Mid (Ct 19-24)**	-	197 (48.9%)	
	**Low (Ct >24)**	-	84 (20.9%)	
	**Unknown**	-	16 (3.9%)	

Table contains the number and % of COVID-19 and non-COVID-19 patients according to sex, age and viral load.[a] Fisher's exact; [b] t-Student test; [c] Chi-square test.

SUPPLEMENTARY FIGURE S1

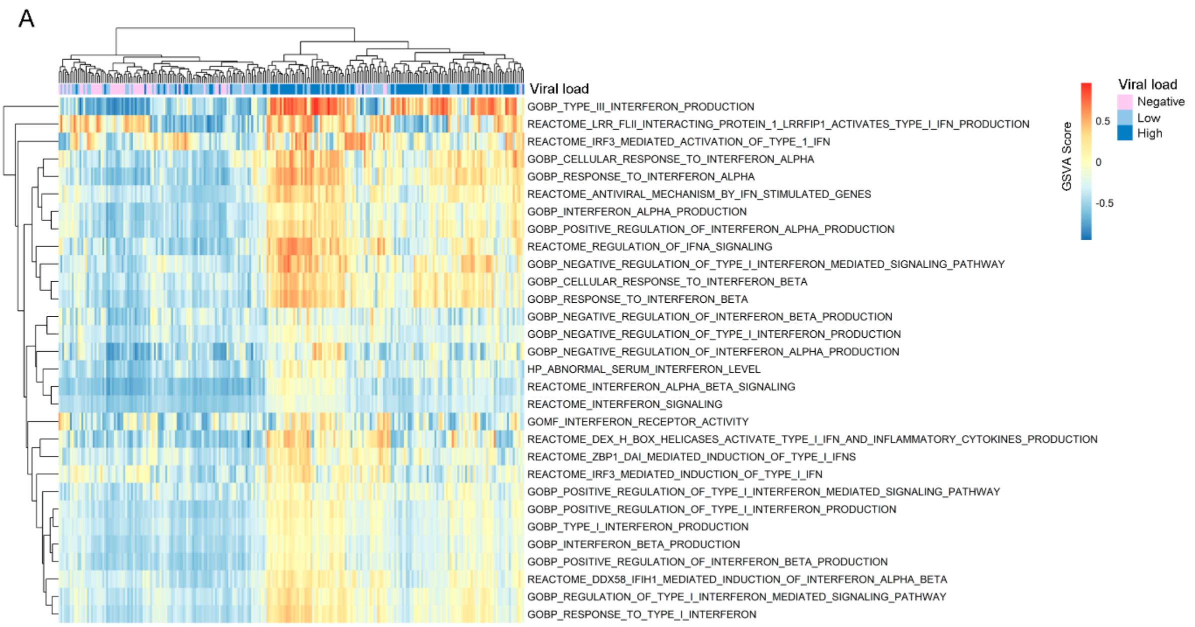

SUPPLEMENTARY FIGURE S1. Global assessment at the transcriptional level of pathways and immune cell types related to IFN-I and IFN-III in COVID-19 positive and negative patients. A) Non-supervised clustering of patients according to their GSVA score in IFN-I and IFN-III genesets. Higher GSVA scores indicate higher activity of the geneset at the RNA level. Each column is labeled according to the COVID-19 viral load of each patient. Viral load is represented as a color scale and was categorized as Negative (pink), Low (first quartile; light blue), or High (fourth quartile; blue). COVID-19 patients with intermediate viral load were excluded from the analysis.

SUPPLEMENTARY FIGURE S2

A

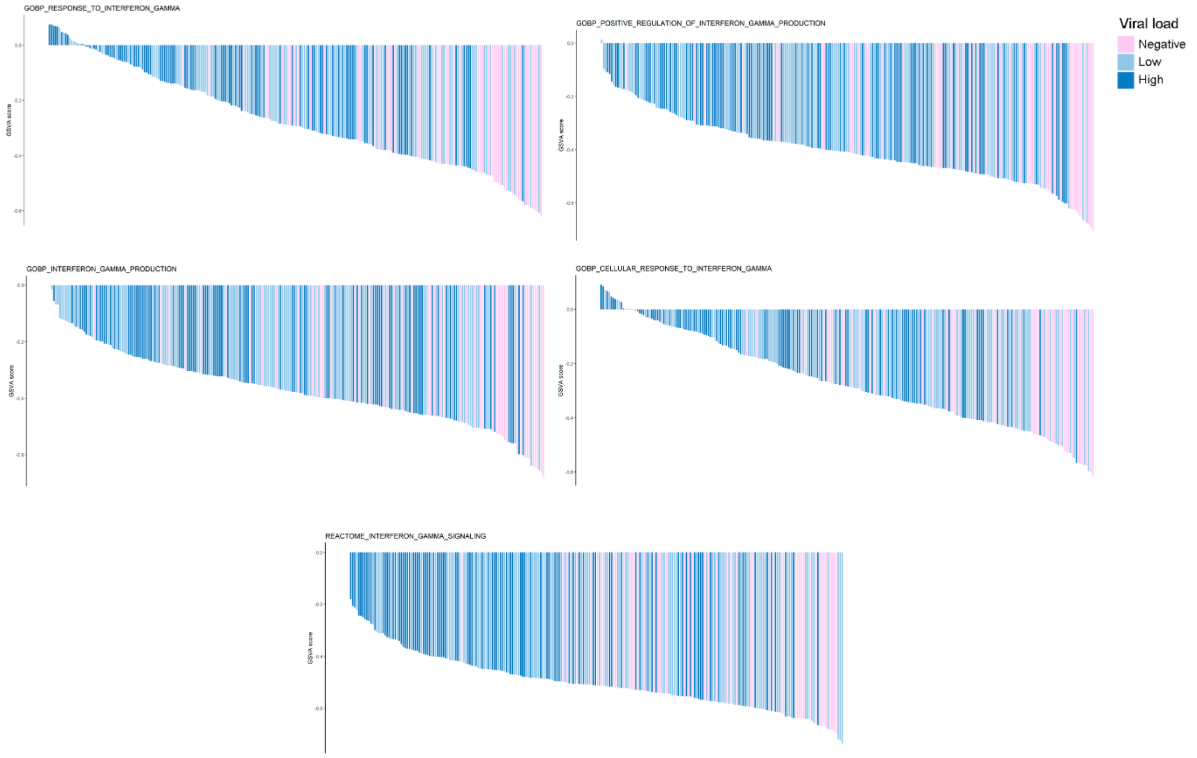

SUPPLEMENTARY FIGURE S2. Global assessment at the transcriptional level of pathways related to IFN-γ production, signaling and regulation of response in COVID-19 positive and negative patients. Waterfall plots of selected genesets that were activated in COVID-19 patients vs. non-COVID-19 patients. Patients are ordered from the highest to the lowest GSVA score in each gene-set.

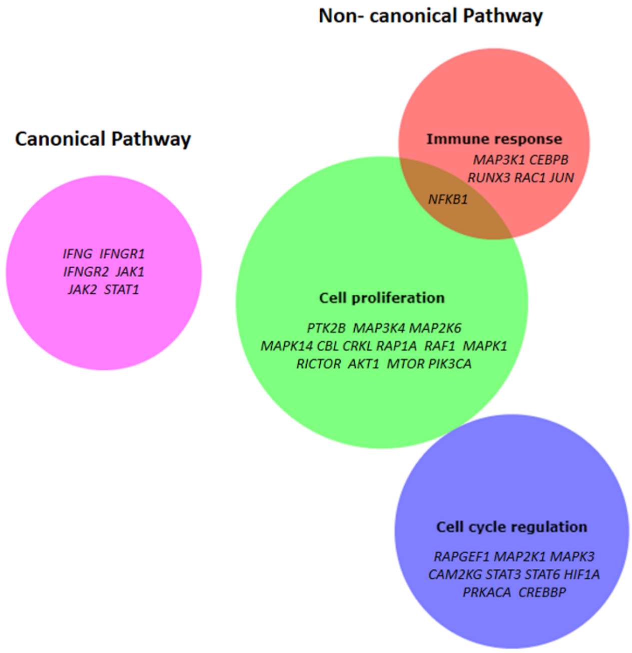

SUPPLEMENTARY FIGURE S3. IFN-γ associated genes. Venn diagram showing IFN-γ associated genes classification. Genes were classified into two categories: genes belonging to the canonical pathway and genes belonging to the non-canonical pathways. IFN-γ associated genes that belong to the non-canonical pathways are either related to immune response, cell proliferation and/or cell cycle regulation.

SUPPLEMENTARY FIGURE S4

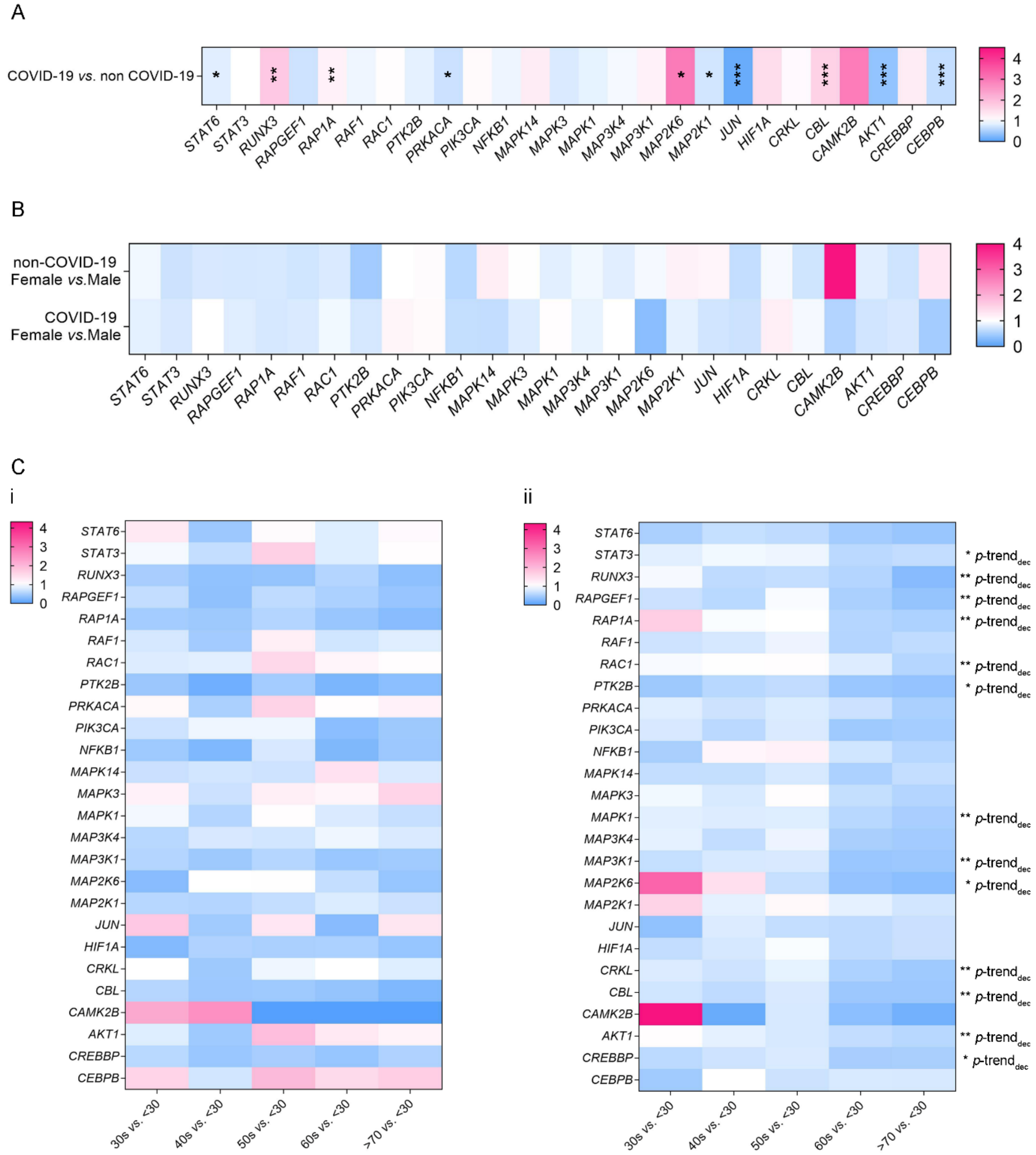

SUPPLEMENTARY FIGURE S4. Expression of genes belonging to the non-canonical IFN-γ pathway in non-COVID-19 and COVID-19 patients from the GSE152075 dataset. Heatmaps depicting the fold change (high = pink; low = blue) for gene expression of genes belonging to the non-canonical IFN-γ pathway between COVID-19 vs. non-COVID-19 patients (A); and female vs. male (B), and age groups 30s, 40s, 50s, 60s & 70s vs. <30 (C) in non-COVID-19 (i) and COVID-19 (ii) patients, assessed by RNA-seq. For (A) & (B) p-values correspond to Wilcoxon rank-sum test, for (C) p-values correspond to decreasing Jonckheere-Terpstra trend test. Statistical significance $*p < 0.05$; $**p < 0.01$; $***p < 0.001$.

SUPPLEMENTARY FIGURE S5

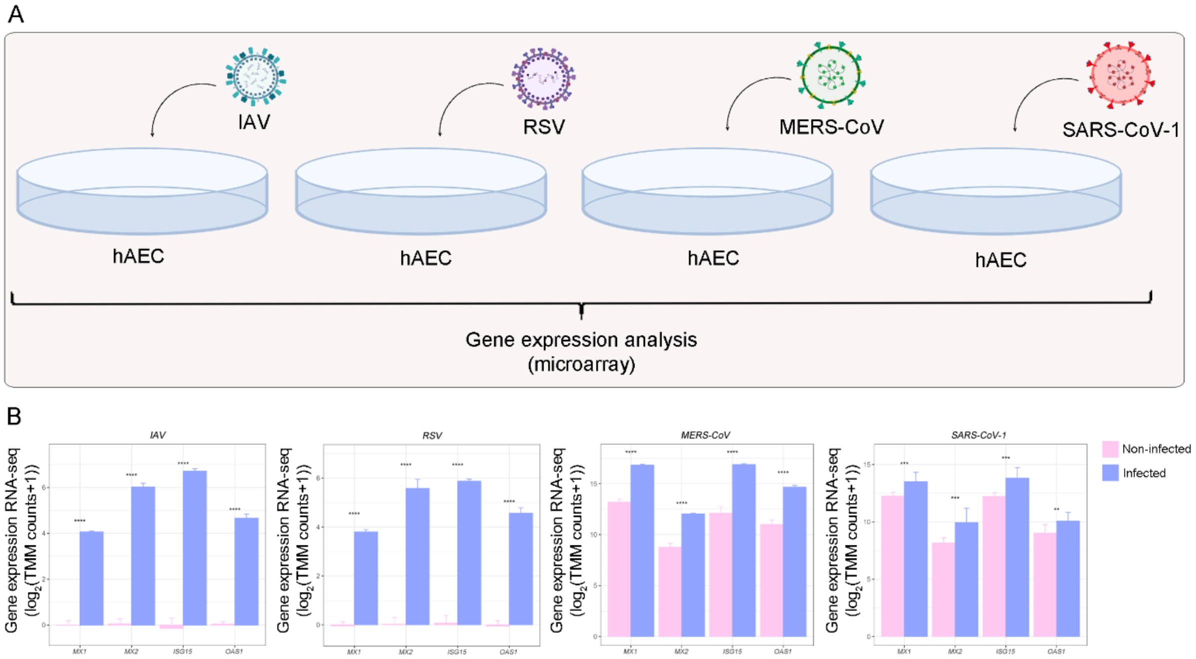

SUPPLEMENTARY FIGURE S5. MX1, MX2, ISG15 and OAS1 expressions in human primary airway epithelial cells (hAEC) that were infected with influenza A (IAV) (2×105 PFU, 24 h), respiratory syncytial virus (RSV) (1×106 PFU, 48 h), Middle East respiratory Syndrome (MERS-CoV) (MOI 5, 48 h) or SARS-CoV-1 (MOI 2, 48 h). A) Schematic representation of the experimental design. B) MX1, MX2, ISG15 and OAS1 expressions in infected (purple) vs. mock-treated (pink) hAEC human cell lines, assessed by microarrays. Data analyzed for IAV and RSV were obtained from the GSE32138 dataset (n = 8). Data analyzed for SARS-CoV-1 and MERS-CoV were obtained from the GSE47963 (n = 20) and GSE100504 (n = 10) datasets, respectively. Student's t test was performed to determine statistical differences. Statistical significance *$p < 0.05$; **$p < 0.01$; ***$p < 0.001$; ****$p<0.0001$.
